# Multi‐Institutional Analysis of Survival and Recurrence Patterns of Different Pathological Regression Types After Neoadjuvant Chemoradiotherapy or Radiotherapy for Esophageal Squamous Cell Carcinoma

**DOI:** 10.1002/cam4.70676

**Published:** 2025-02-13

**Authors:** Fangdong Zhao, Qifeng Wang, Weiming Han, Wubulaishan Maitudi, Fuliang Cao, Tian Zhang, Xi Chen, Jie Dong, Lei Gong, Xiaobin Shang, Hongjing Jiang, Wencheng Zhang, Qingsong Pang, Zefen Xiao, Ping Wang, Peng Tang

**Affiliations:** ^1^ Department of Radiation Oncology Tianjin Medical University Cancer Institute & Hospital, National Clinical Research Center for Cancer, Key Laboratory of Cancer Prevention and Therapy, Tianjin's Clinical Research Center for Cancer Tianjin China; ^2^ Department of Radiation Oncology, Sichuan Cancer Hospital and Institution, Sichuan Cancer Center, School of Medicine University of Electronic Science and Technology of China, Radiation Oncology Key Laboratory of Sichuan Province Chengdu China; ^3^ Department of Radiation Oncology National Cancer Center/National Clinical Research Center for Cancer/Cancer Hospital, Chinese Academy of Medical Sciences and Peking Union Medical College Beijing China; ^4^ Department of Endoscopy Diagnosis and Therapy Tianjin Medical University Cancer Institute and Hospital, National Clinical Research Center for Cancer, Key Laboratory of Cancer Prevention and Therapy, Tianjin's Clinical Research Center for Cancer Tianjin China; ^5^ Department of Nutrition Therapy Tianjin Medical University Cancer Institute and Hospital, National Clinical Research Center for Cancer, Key Laboratory of Cancer Prevention and Therapy, Tianjin's Clinical Research Center for Cancer Tianjin China; ^6^ Department of Esophageal Cancer Tianjin Medical University Cancer Institute and Hospital, National Clinical Research Center for Cancer, Key Laboratory of Cancer Prevention and Therapy, Tianjin's Clinical Research Center for Cancer Tianjin China

**Keywords:** esophageal squamous cell carcinoma, neoadjuvant chemoradiotherapy, pathological regression, recurrence, ypT0N+

## Abstract

**Background:**

The recurrence patterns of different types of pathologic regression of the primary tumor and lymph nodes in patients with esophageal squamous cell carcinoma (ESCC) after neoadjuvant chemoradiotherapy (NCRT) are little known, especially in ypT0N+ patients.

**Methods:**

We included 582 patients with ESCC who had esophagectomy after NCRT or neoadjuvant radiotherapy (NRT) from 3 institutions. The patients were divided into 4 groups: ypT0N0, ypT0N+, ypT+N0, and ypT+N+ according to the type of pathological regression of the primary tumor and lymph nodes. Survival, recurrence pattern and timing, and potential prognostic factors were compared.

**Results:**

A total of 179 patients were classified as ypT0N0, 227 patients as ypT + N0, 45 patients as ypT0N+, and 131 patients as ypT + N+. The median follow‐up was 31.7 months in all patients. The restricted mean survival time (RMST) of ypT0N0, ypT + N0, ypT0N+, and ypT + N+ patients decreased sequentially (70.64, 63.84, 55.93 and 39.96 months) and the recurrence rates increased sequentially (22.3%, 29.5%, 44.4% and 54.2%). Both the overall survival (OS) and recurrence‐free survival (RFS) in the ypT0N+ group were significantly lower than those in the ypT0N0 group (HR: 2.226, *p* = 0.007; HR: 2.271, *p* = 0.003). The distant metastasis (DM) pattern in ypT0N+ was similar to that of ypT + N+, and higher than that of ypN0 (25.6% vs 14.3%, HR: 1.970, *p* = 0.040).

**Conclusions:**

ESCC patients with various pathological regression types after receiving NCRT or NRT had significantly different survival rates. ypT0N+ patients had a lower survival rate and higher DM rate than ypT0N0 patients. For these lymph node‐positive patients, adjuvant chemotherapy does not appear to improve their prognosis.

## Graphical abstract

We included ESCC patients who had received neoadjuvant therapy from three major academic institutions and provided the largest dataset of recurrence pattern data for ypT0N+ patients. Survival, recurrence pattern and timing, and potential prognostic factors were compared. Significant survival differences existed among patients with various pathological regression types who received NCRT or NRT and surgery for ESCC. The ypT0N+ group had a poorer survival rate. Besides, the distant metastasis rate in ypT0N+ was higher than that in the ypT0N0 group and similar to that in the ypT+N+ group. For these lymph node‐positive patients, adjuvant chemotherapy does not appear to improve their prognosis.

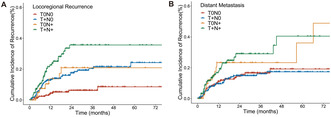

## 1 Introduction

Esophageal cancer ranks as the sixth leading cause of cancer‐related mortality worldwide [1]. Neoadjuvant chemoradiotherapy (NCRT) is the standard treatment for patients with resectable locally advanced esophageal cancer [2, 3]. Several clinical trials, including Cross and NEOCRTEC5010, have demonstrated that adding NCRT to surgery significantly improves patient prognosis compared with surgery alone [4, 5]. However, the prognosis for locally advanced esophageal cancer is still poor, with a 5‐year overall survival (OS) rate of less than 50% for NCRT followed by surgery [6]. The primary causes of death in esophageal cancer patients are recurrence and metastasis. Furthermore, the histopathological response following NCRT, the presence or absence of residual tumor in the resected specimen, is an essential factor affecting recurrence and survival [7].

A recent study found that the response to NCRT was closely associated with improved survival in patients with esophageal cancer [8]. Patients could be classified into four pathological regression types according to the residual status of primary tumor lesions (ypT0/ypT+) and lymph nodes (ypN0/ypN+) after NCRT. Several studies indicated that these pathological regression types were differently associated with patient survival. Patients with pathologic complete response (pCR) (ypT0N0) had significantly better long‐term survival compared with near‐complete and partial responses [9]. Patients with pCR had lower recurrence rates and better survival than incomplete responders [10]. However, the prognostic characteristics of the four specific pathological regression types are undefined. Therefore, it is necessary to further define the survival and recurrence patterns of patients with different types of pathologic response, which will contribute to the improvement of clinical management strategies.

Patients with ypT0N+ who achieved complete response in primary tumors and residual lymph nodes had a low prevalence. Limited studies are available regarding their survival and recurrence patterns, with divergent outcomes from different centers [11, 12]. Some studies suggested their survival was similar to that of ypT0N0 patients, while others suggested that their survival was worse. The survival significance and recurrence pattern of ypT0N+ in relation to ypT0N0 remain unclear. Our results would benefit from optimizing postoperative treatment strategies for patients with different responses after NCRT.

## 2 Methods

### 2.1 Patient Selection

This retrospective study included 582 patients with esophageal squamous cell carcinoma (ESCC) who received NCRT or neoadjuvant radiotherapy (NRT) followed by esophagectomy from 2003 to 2022. The patients were recruited from three academic cancer institutions: Tianjin Cancer Hospital, Cancer Hospital of the Chinese Academy of Medical Sciences, and Sichuan Cancer Hospital. The inclusion criteria includedhistologically confirmed potentially resectable ESCC, R0 resection, and clinical stage T1‐4N0‐1M0 (stage I–III) according to the American Joint Committee on Cancer 6th edition staging criteria [13]. Exclusion criteria includedincomplete surgical resection, two‐dimensional radiotherapy, and incomplete clinical data. Before the start of treatment, all patients underwent pre‐treatment evaluation, consisting of laboratory tests (including hematology and biochemistry), physical examination, esophagogastroduodenoscopy (EGD) combined with endoscopic ultrasound (EUS) and biopsy, and chest/abdominal computed tomography (CT) and/or positron emission tomography (PET). The study was approved by the institutional review boards of the participating institutions.

### 2.2 Treatment

All patients received NRT with external beam irradiation using three‐dimensional conformal radiotherapy or intensity‐modulated radiotherapy. The standard radiotherapy regimen was 40 Gy in 2.0 Gy daily fractions and 5 fractions per week. A total of 535 (91.9%) patients received paclitaxel with platinum or fluorouracil neoadjuvant chemotherapy during radiotherapy. All patients received preoperative reassessment 4–8 weeks after the end of neoadjuvant therapy. Based on the tumor location, most patients received the McKeown procedure, and a subset of patients received the Ivor‐Lewis procedure or minimally invasive esophagectomy. All patients received at least two‐field lymphadenectomy, with three‐field lymph node dissection for patients with suspected or confirmed supraclavicular lymph node metastases. The resected primary tumor lesions and lymph nodes were evaluated by H.E staining by professional pathologists. Based on the presence or absence of residual tumor cells in the primary tumor lesion, the specimens were classified as T0 (complete regression of the tumor in the primary lesion) and T+ (residual tumor cells remaining in the primary lesion). Similarly, according to the presence or absence of residual tumor cells in the resected lymph nodes, the specimens were classified into N0 (no positive lymph nodes) and N+ (positive lymph nodes). Then, by combining the residual tumors in both the resected primary tumor lesion and lymph nodes, the patients were divided into four groups: ypT0N0, ypT+N0, ypT0N+, and ypT+N+.

### 2.3 Surveillance and Recurrences

All patients were followed up every 3 months during the initial 2 years after surgery, every 6 months thereafter, and then annually at least after 5 years. Follow‐up evaluation included physical examination, laboratory tests, CT or PET CT of the chest and abdomen, upper gastrointestinal contrast, and ultrasound of the neck and abdomen. Some patients with suspected progression require further endoscopy or ultrasonography and pathological examination to clarify the diagnosis. Recurrence was categorized into locoregional recurrence (LRR) and distant metastasis (DM) according to the site of the first progression. LRR was defined as recurrence occurring in the anastomosis, esophagus, or tumor bed and in the regional lymph nodes. DM was defined as metastasis to non‐regional lymph nodes and other hematogenous organisms.

### 2.4 Statistical Analysis

Continuous variables were described using medians and means. Rank or categorical variables were described as counts and percentages. Comparisons of categorical variables were performed by chi‐square or Fisher's exact test.

OS times were calculated as the time from the date of surgery to death or last follow‐up; recurrence‐free survival (RFS) was defined as the time from the date of surgery to the first disease recurrence. OS, RFS, LRR rate, and DM rate were analyzed by the Kaplan–Meier method, and the log‐rank test was used to test the differences among groups. Restricted mean survival time (RMST) analysis was also used to provide a more intuitive and clearer description of the survival of each group by calculating the mean survival time within a specific cut‐off time, and the difference in the mean survival time between the groups was calculated to assess the difference in survival between the different groups [14, 15]. Univariable and multivariable Cox proportional hazards regression models were used to analyze the effects of different factors on OS and RFS. The covariates with *p* < 0.1 in the univariable analysis were entered into the multivariable analysis. Univariable Cox regression models were used to analyze the between‐group differences in recurrence patterns in separate recurrence sites. Propensity score matching (PSM) was used to equalize between‐group differences. Subgroup analysis of adjuvant therapy efficacy used multivariable Cox regression. Statistical calculations were performed using SPSS 25, and survival curves were plotted using R 4.2.2.

## 3 Result

### 3.1 Patient Characteristics

A total of 582 patients from 3 institutions (134 from Tianjin Medical University Cancer Institute and Hospital, 175 from Cancer Hospital Chinese Academy of Medical Sciences, and 273 from Sichuan Cancer Hospital) were included in the study. According to residual tumors in the resected primary tumor lesion and lymph nodes, the patients were divided into four groups: 179 patients (30.8%) in the ypT0N0 group, 227 patients (39%) in the ypT + N0 group, 45 patients (7.7%) in the ypT0N+ group, and 131 patients (22.5%) in the ypT + N+ group. The median age of the whole cohort was 60 years (range 42–76 years), with 87.1% male. There were no significant differences in basic patient characteristics, such as age, sex, clinical stage, and radiation dose, among the four groups (Table 1). While the ypT + N0 group had more patients not receiving neoadjuvant chemotherapy. Notably, a total of 60 patients had preoperative biopsy pathological results. Patients with no residual tumor cells in the biopsy pathology were classified as complete response (CR), with 27 patients in this group. Patients with tumor cells present in the biopsy pathology were classified as having residual disease, with 33 patients in this group. Only 74.1% of the patients with CR indicated by preoperative biopsy were confirmed to have a complete pathological response in the primary lesion postoperatively. In 25.9% of patients, despite preoperative biopsy suggesting complete tumor regression, postoperative pathology still revealed residual tumor in the primary lesion (Figure S1).

**TABLE 1:** Patient characteristics.

Characteristics	ypT0N0	ypT+N0	ypT0N+	ypT+N+	*p*
	(*n* = 179) %	(*n* = 227) %	(*n* = 45) %	(*n* = 131) %	
Age					
< 60	82 (45.8)	109 (48.0)	26 (57.8)	73 (55.7)	0.22
≥ 60	97 (54.2)	118 (52.0)	19 (42.2)	58 (44.3)	
Sex					
Male	150 (83.8)	197 (86.8)	39 (86.7)	121 (92.4)	0.172
Female	29 (16.2)	30 (13.2)	6 (13.3)	10 (7.6)	
Tumor location					
Upper	33 (18.4)	47 (20.7)	8 (17.8)	13 (9.9)	0.022
Middle	86 (48.1)	88 (38.8)	13 (28.9)	58 (44.3)	
lower	60 (33.5)	92 (40.5)	24 (53.3)	60 (45.8)	
Clinical T stage					
T1‐2	11 (6.1)	13 (5.7)	2 (4.4)	8 (6.1)	0.505
T3	122 (68.2)	132 (58.1)	29 (64.5)	81 (61.8)	
T4	46 (25.7)	82 (36.1)	14 (31.1)	42 (32.1)	
Clinical N stage					
N0	14 (7.8)	22 (9.7)	2 (4.4)	9 (6.9)	0.6
N1	165 (92.2)	205 (90.3)	43 (95.6)	122 (93.1)	
Clinical TNM stage					
IIA‐IIB	18 (10.1)	23 (10.1)	4 (8.9)	13 (9.9)	0.872
III	156 (87.1)	202 (89.0)	41 (91.1)	116 (88.5)	
IVA‐IVB	5 (2.8)	2 (0.9)	0 (0)	2 (1.5)	
Radiation dose					
≤ 40Gy	127 (70.9)	175 (77.1)	36 (80.0)	106 (80.9)	0.187
> 40Gy	52 (29.1)	52 (22.9)	9 (20.0)	25 (19.1)	
Chemotherapy					
Yes	171 (95.5)	200 (88.1)	44 (97.8)	120 (91.6)	0.022
No	8 (4.5)	27 (11.9)	1 (2.2)	11 (8.4)	
Number of LN examined					
< 10	17 (9.5)	32 (14.1)	1 (2.2)	10 (7.6)	0.052
≥ 10	162 (90.5)	195 (85.9)	44 (97.7)	121 (92.4)	

Abbreviation: LN, lymph nodes.

### 3.2 Survival Outcome

**FIGURE 1:**
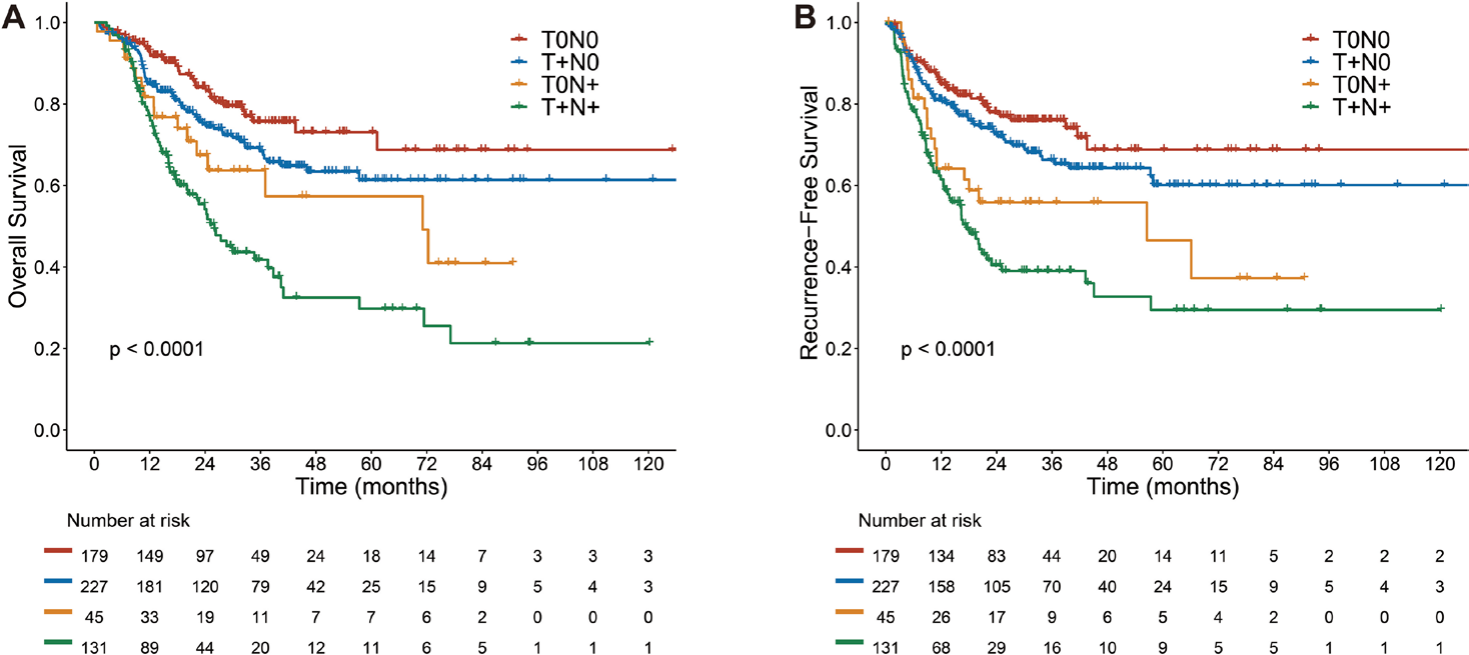
Overall survival (A) and recurrence‐free survival (B) of esophageal squamous cell carcinoma patients with different pathologic regression types.

The median follow‐up was 31.7 months (range, 3.1–162.8 months) for the whole cohort. The median follow‐up was 30.8, 36.4, 26.9, and 30.4 months in the ypT0N0, ypT + N0, ypT0N+, and ypT + N+ groups, respectively. Follow‐up times among groups were not statistically different. The 5‐year OS rate and 5‐year RFS rate were 57.5% (95% CI, 52.2–63.3) and 54.6% (95% CI, 48.9–61.0), respectively. The median OS time and RFS time were not reached for the whole cohort. We found there were significant differences in OS and RFS among the four groups (*p* < 0.001) (Figure 1; Table S1). The patients in the ypT0N+ group had a shorter OS than those in the ypT0N0 group (HR: 2.226, 95% CI, 1.243–3.987, *p* = 0.007; Figure 1A). RFS showed the same trend, with a significantly lower RFS in the ypT0N+ group versus the ypT0N0 group (HR: 2.271, 95% CI, 1.327–3.886, *p* = 0.003; Figure 1B). No statistically significant difference was found in RFS between the ypT0N+ group and the ypT + N+ group (HR:0.696, 95% CI: 0.423–1.143; *p* = 0.143). The 5‐year OS rates were 73.1% (95% CI, 64.5–82.7), 61.4% (95% CI, 53.5–70.4), 57.3% (95% CI, 41.5–79.2), and 29.8% (95% CI, 20.2–44.0) in the ypT0N0, ypT + N0, ypT0N+, and ypT + N+ groups, respectively. The 5‐year RFS rates in the four groups were 68.8% (95% CI, 59.4–79.7), 60.1% (95% CI, 51.7–69.8), 46.5% (95% CI, 29.6–73.1) and 29.5% (95% CI, 19.6–44.2), respectively (Table S1).

**FIGURE 2:**
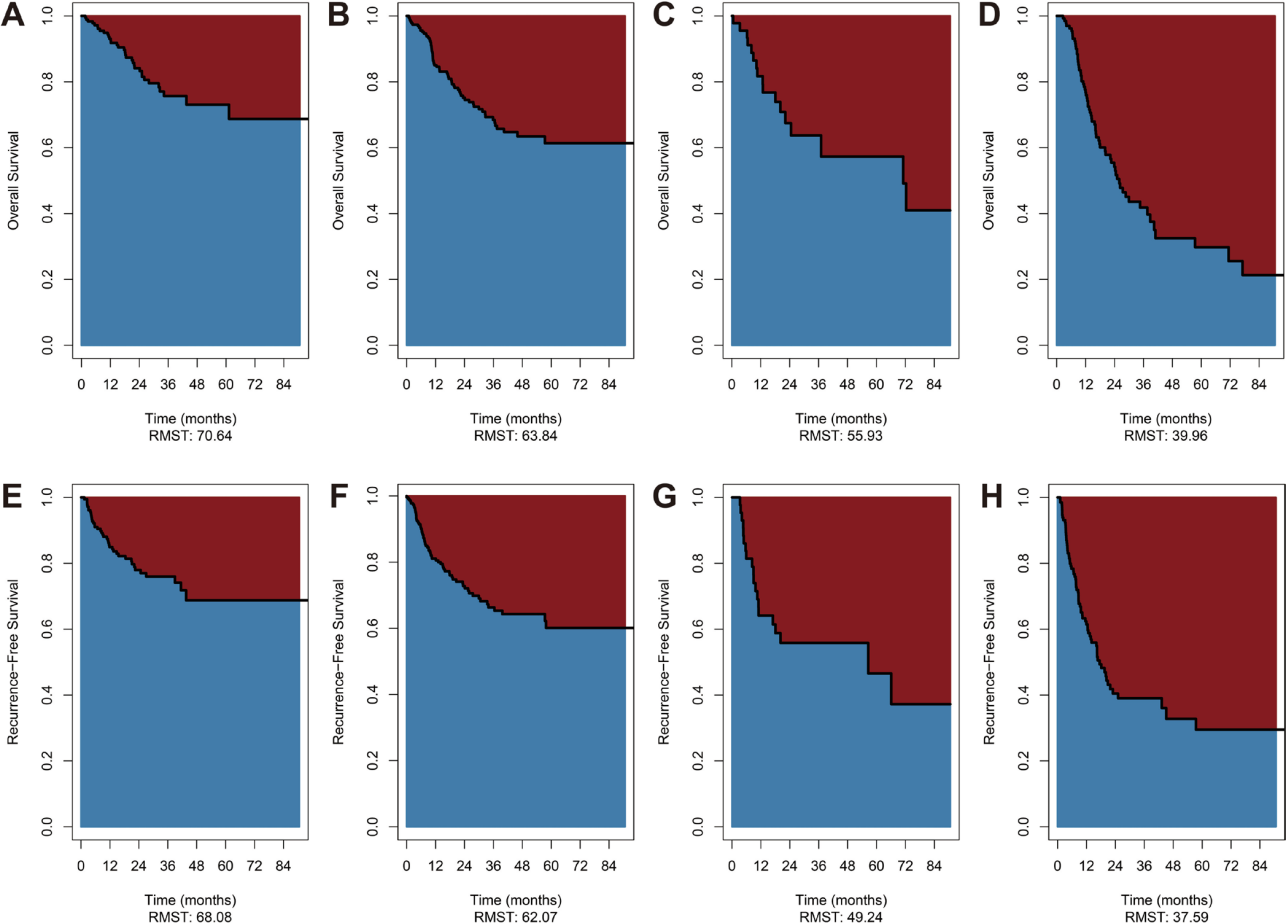
Restricted mean overall survival time and restricted mean recurrence‐free survival time of esophageal squamous cell carcinoma patients with different pathologic regression types. (1)Restricted mean overall survival time for patients with ypT0N0 (A), ypT+N0 (B), ypT0N+ (C), ypT+N+ (D). (2) Restricted mean recurrence‐free survival time for patients with ypT0N0 (E), ypT+N0 (F), ypT0N+ (G), ypT+N+ (H).

In addition, RMST uses the mean survival time to visualize the difference in survival between groups more clearly. The RMST of OS was 55.93 months in the ypT0N+ group and 70.64 months in the ypT0N0 group (RMST difference, 14.71 months, 95% CI: 0.7955–28.623, *p* = 0.038) (Figure 2A,C). The RMST of RFS was 49.24 months in the ypT0N+ group and 68.08 months in the ypT0N0 group, with a statistically significant difference between the two groups (RMST difference, 18.84 months, 95% CI: 4.609–33.072, *p* = 0.009) (Figure 2E,G). The RMST of RFS in the ypT + N+ group was 37.59 months (RMST difference with ypT0N+, 11.65 months, *p* = 0.126) (Table 2).

**TABLE 2:** RMST difference for OS and RFS.

	Overall survival		Recurrence‐free survival	
	RMST difference (95% CI)	*p*	RMST difference (95% CI)	*p*
T0N0 vs. T0N+	14.709 (0.795 ~ 28.623)	0.038	18.841 (4.609 ~ 33.072)	0.009
T+N+ vs. T0N+	−15.971 (−30.359 ~ −1.582)	0.030	−11.651 (−26.562 ~ 3.260)	0.126
T+N0 vs. T0N+	7.913 (−5.662 ~ 21.489)	0.253	12.830 (−1.071 ~ 26.732)	0.070
T+N0 vs. T0N0	−6.795 (−14.942 ~ 1.352)	0.102	−6.010 (−14.546 ~ 2.526)	0.168

Abbreviations: CI, confidence interval; OS, overall survival; RFS, recurrence‐free survival; RMST, restricted mean overall survival time.

### 3.3 Recurrence Characteristics of Whole Cohort

A total of 198 patients (34.0%) experienced recurrence. The recurrence rate was 22.3% (40/179), 29.5% (67/227), 44.4% (20/45) and 54.2% (71/131) in the ypT0N0, ypT+N0, ypT0N+, and ypT+N+ groups, respectively (Table 3). The median time from surgery to the first recurrence was 8.6 months (interquartile range [IQR], 4.2–16.3 months). A total of 88.4% (175/198) of recurrences occurred within 2 years after surgery and 93.9% (186/198) occurred within 3 years of surgery. N+ patients had a relatively early recurrence time compared to N0 patients. Only 6.6% (6/91) of recurrences occurred 2 years postoperatively in N+ patients, whereas 15.9% (17/107) of recurrences occurred for N0 patients (*p* = 0.047) (Figure S2) Additionally, the recurrence rate was significantly higher in N+ patients compared with that in N0 patients (51.7% vs 26.4%, *p* < 0.001).

**TABLE 3:** Recurrence patterns and sites.

Recurrence type	ypT0N0 *n* = 174 (%)	ypT+N0 *n* = 218 (%)	ypT0N+*n* = 43 (%)	ypT+N+*n* = 120 (%)	Total recurrence *n* = 172 (%)
Esophagus	5 (2.9)	13 (6.0)	1 (2.3)	9 (7.5)	28 (16.3)
Regional LN	8 (4.6)	28 (12.8)	6 (13.9)	25 (20.8)	67 (38.9)
Locoregional	10 (5.7)	39 (17.9)	7 (16.3)	33 (27.5)	89 (51.7)
Distant	26 (14.9)	30 (13.8)	11 (25.6)	29 (24.2)	96 (55.8)
Lung	12 (6.9)	14 (6.4)	2 (4.6)	15 (12.5)	43 (25.0)
Liver	9 (5.2)	7 (3.2)	4 (9.3)	6 (5.0)	26 (15.1)
Bone	6 (3.4)	5 (2.3)	2 (4.6)	11 (9.2)	24 (13.9)
Brain	2 (1.1)	5 (2.3)	1 (2.3)	1 (0.8)	9 (5.2)
Retroperitoneal LN	1 (0.6)	2 (0.9)	2 (4.6)	3 (2.5)	8 (4.6)
Pleura	0 (0)	4 (1.8)	1 (2.3)	1 (0.8)	6 (3.5)
Adrenal	1 (0.6)	0 (0)	1 (2.3)	1 (0.8)	3 (1.7)
Other locations	1 (0.6)	2 (0.9)	2 (4.6)	3 (2.5)	8 (4.6)

Abbreviation: LN, lymph nodes.

### 3.4 Recurrence Patterns Among Groups

We then analyzed the site of recurrence in 172 patients because 27 patients were excluded from the total 198 patients with recurrence due to unknown recurrence sites. 89 (51.7%) patients had LRR, and 96 (55.8%) had DM (Table 3).

**FIGURE 3:**
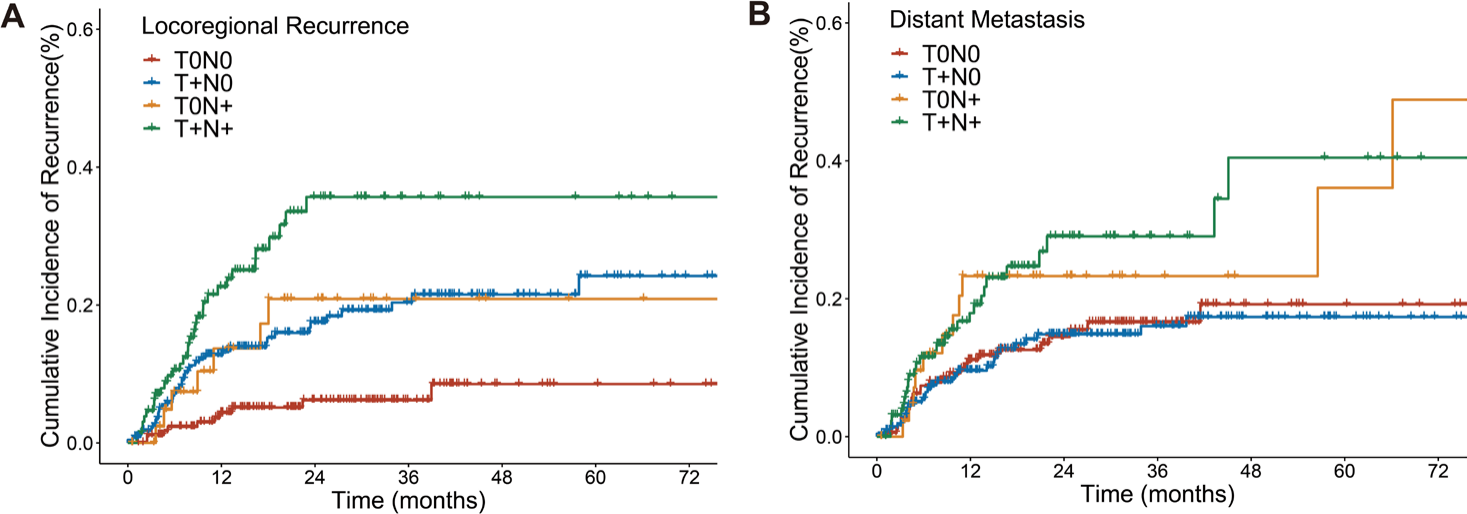
Locoregional failure‐free survival (A) and distant metastasis‐free survival (B) of esophageal squamous cell carcinoma patients with different pathologic regression types.

Between‐group comparisons revealed variations in the recurrence patterns among the four groups. We first compared the LRR rates among the four groups. The ypT0N+ group had more LRR patients compared with the ypT0N0 group (16.3% vs 5.7%, HR: 3.198, 95% CI, 1.217–8.404; *p* = 0.018). There was no significant difference in LRR occurrence between ypT0N+ with ypT+N0 or ypT+N+ group (16.3%, 17.9% and 27.5%, respectively) (Figure 3A). We then analyzed the DM patterns in the four groups. Similar to our findings in the LRR analysis, the ypT0N+ group showed a higher DM rate than the ypN0 groups (25.6% vs 14.3%, HR: 1.970, 95% CI, 1.032–3.761; *p* = 0.040). Of the ypN0 groups, the ypT0N+ group was higher than the ypT+N0 group (25.6% vs 13.8%, HR: 2.039, 95% CI, 1.021–4.070; *p* = 0.043) and showed a higher tendency than the ypT0N0 group (25.6% vs 14.9%, HR: 1.890, 95% CI, 0.933–3.826; *p* = 0.077). The ypT0N+ group had a similar DM rate as that in the ypT+N+ group (25.6% vs 24.2%, HR: 0.957, 95% CI, 0.478–1.917; *p* = 0.902). (Figure 3B, Table 4). Additionally, neoadjuvant chemoradiotherapy only became the standard treatment after the publication of the CROSS trial results. Therefore, we analyzed the survival outcomes and recurrence patterns of patients treated after 2012, and the results were consistent with the overall data (Tables [Link], [Link], [Link]; Figure S3–S5). We also analyzed the DM at specific sites. The most frequent locations for DM were the lungs, liver, bones, and brain, in order. The retroperitoneal lymph nodes, pleura, and adrenal glands experienced low DM involvement (Table 3).

**TABLE 4:** Comparison of recurrence patterns in different pathologic regression types.

	Locoregional recurrence		Distant metastasis	
	HR (95% CI)	*p*	HR (95% CI)	*p*
T0N0 vs. T0N+	3.198 (1.217–8.404)	0.018	1.890 (0.933–3.826)	0.077
T0N+ vs. T+N+	1.860 (0.822–4.207)	0.136	1.045 (0.522–2.093)	0.902
T+N0 vs. T0N+	1.007 (0.450–2.252)	0.986	2.039 (1.021–4.070)	0.043
T0N0 vs. T+N0	3.175 (1.585–6.362)	0.001	0.927 (0.548–1.568)	0.776

Abbreviations: CI, confidence interval; HR, hazard ratio.

### 3.5 Prognostic Factors for Survival and Recurrence

Univariable and multivariable analyses of risk factors for survival and recurrence in patients with ESCC who underwent neoadjuvant therapy and subsequent surgery are presented in Table 5. The multivariable analysis revealed that residual positive lymph nodes and a high pathologic T‐stage were independent risk factors for both survival and recurrence. Additionally, the presence of an anastomotic fistula and the radiation dose< 40Gy were predictive of recurrence. The number of lymph nodes (LN) examined < 10 was predictive of survival (Table 5).

**TABLE 5:** Univariate and multivariate analyses for overall survival and recurrence‐free survival.

Variable	Overall Survival			Recurrence‐free survival		
	Univariate	Multivariate		Univariate	Multivariate	
	*p*	HR (95% CI)	*p*	*p*	HR (95% CI)	*p*
Age						
< 60	0.055	1	0.234	0.08	1	0.399
≥ 60		0.837 (0.625–1.122)			0.885 (0.665–1.176)	
Sex						
Male	0.036	1	0.093	0.064	1	0.218
Female		0.624 (0.360–1.081)			0.735 (0.451–1.199)	
Tumor location						
Upper/Middle	0.817			0.91		
lower						
Clinical T stage						
T1‐2	0.525			0.446		
T3‐4						
Clinical N stage						
N0	0.483			0.117		
N1						
Radiation dose						
≤ 40Gy	0.205			0.012	1	0.032
> 40Gy					0.657 (0.447–0.964)	
Chemotherapy						
Yes	0.233			0.663		
No						
Number of LN examined						
< 10	0.03	1				
≥ 10		0.586 (0.384–0.894)	0.013	0.147		
Histologic grade						
GxG1G2	0.027	1				
G3		1.254 (0.919–1.712)	0.153	0.372		
Positive lymph node						
0	< 0.001	1			1	< 0.001
> 0		2.218 (1.635–3.008)	< 0.001	< 0.001	2.206 (1.644–2.960)	
ypT stage						
T0–2		1			1	
T3	< 0.001	1.944 (1.276–2.963)	0.002	0.001	1.633 (1.046–2.552)	0.031
T4	< 0.001	4.098 (1.927–8.715)	< 0.001	< 0.001	6.730 (3.208–14.119)	< 0.001
T downstage						
Yes	0.001	1			1	0.306
No		1.379 (0.858–2.217)	0.185	< 0.001	1.296 (0.789–2.128)	
Vessel carcinoma embolus						
Yes	0.002	1			1	0.385
No		1.341 (0.800–2.246)	0.265	0.005	1.296 (0.789–2.128)	
Anastomotic fistula						
Yes	0.336			0.017	1	0.014
No					0.408 (0.199–0.835)	

Abbreviations: CI, confidence interval; HR, hazard ratio; LN, lymph nodes.

### 3.6 Subgroup Analysis of Adjuvant Therapy

**FIGURE 4:**
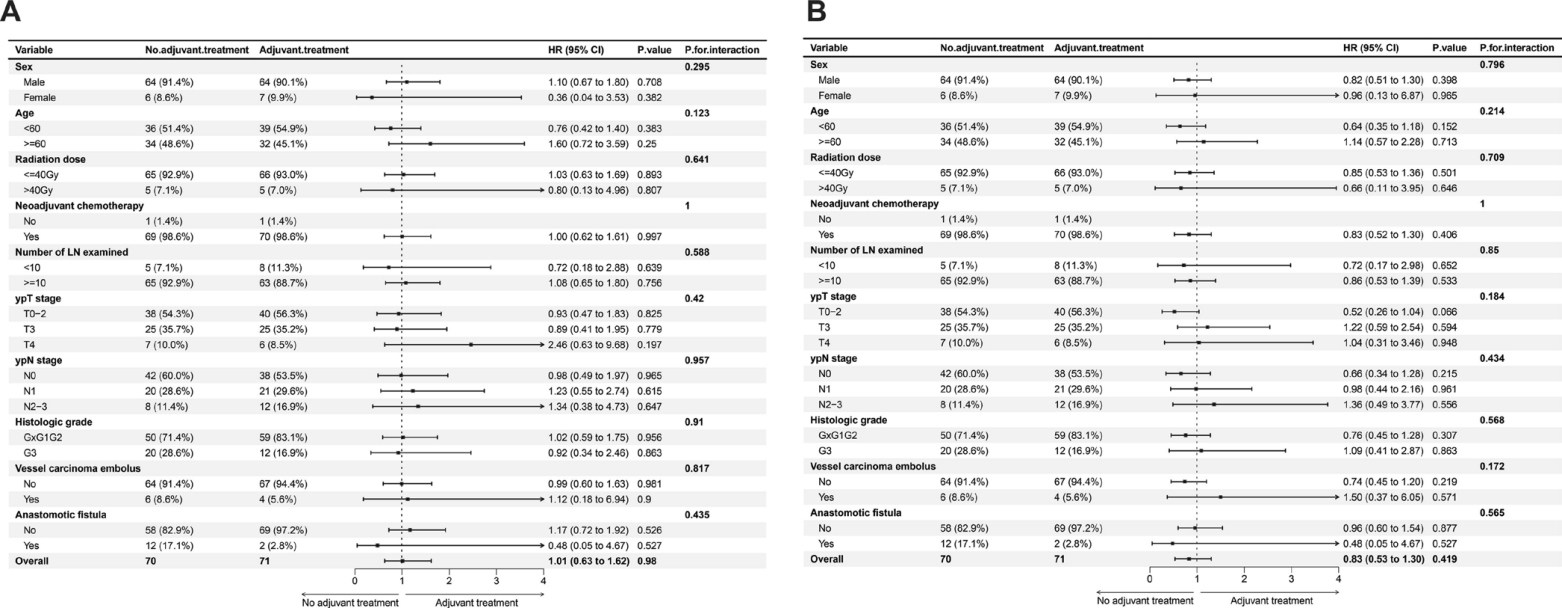
Forrest plot of the effect of adjuvant treatment on the hazard ratio for death (A) and recurrence (B).

In the previous analysis, we observed a significantly worse prognosis in patients with ypN+. To investigate whether adjuvant therapy could improve patient prognosis, we conducted an in‐depth analysis. A total of 71 patients underwent platinum‐based adjuvant chemotherapy. After 1:1 PSM, 141 patients were included in the subgroup analysis of adjuvant therapy. The baseline characteristics are shown in (Table S5). The results showed that postoperative adjuvant therapy did not significantly affect the prognosis in terms of either OS or RFS across all subgroups (Figure 4). Therefore, postoperative adjuvant chemotherapy may not improve the prognosis of patients with persistent lymph node positivity after surgery.

## 4 Discussion

The important impact of primary or lymph node pathological residual after neoadjuvant therapy on patient prognosis has been widely acknowledged. However, the recurrence patterns of patients with different types of pathological regression of the primary tumor and lymph nodes remain unclear. In the multicenter study, we found significant differences in survival and recurrence patterns for four pathological regression types in ESCC patients receiving NCRT or NRT and surgery. In particular, the ypT0N+ patients had worse survival and more DM, even though the primary lesion was in complete regression. To the best of our knowledge, this is the largest data for patients with pathology type ypT0N+ in analyzing the recurrent patterns after undergoing neoadjuvant therapy and esophagectomy.

In the current study, poor regression of the primary lesion (ypT) and lymph node positivity (ypN) were independent risk factors for survival in patients with ESCC. Independent risk factors for survival in ESCC patients who underwent surgery after neoadjuvant therapy remain controversial. Reynolds JV et al. [16] study showed that ypN was the most powerful determinant of survival in patients with esophageal cancer, whereas tumor regression grade (TRG) or ypT status was not associated significantly with prognosis. On the contrary, the regression status of ypT significantly affected prognosis [17, 18]. Furthermore, Paul M et al. [19] reported that both primary tumor regression and lymph node status (ypN) were important prognostic indicators for patients with complete resections (R0) after neoadjuvant chemoradiotherapy for esophageal cancer, which was consistent with our findings. The study by Yun JK et al. [20] combined ypT with ypN and compared the survival of three groups, high (ypT0N0), mid (ypT0N+ or ypT + N0), and low (ypT + N+) groups, which showed a gradual decline in survival and better discriminatory and more accurate predictive ability using a combined assessment system of ypT and ypN. However, this study did not focus on the survival characteristics of groups ypT + N0 and ypT0N+. This current study combined ypT and ypN to describe the survival of four pathological regression types in more detail.

We explored survival by applying RMST for OS analysis. RMST calculated the area under the KM curve and mean survival time for a defined follow‐up time when quantifying differences between groups, providing more intuitive results for comparison. McCaw ZR et al. [21] suggested that relying solely on median survival might lead to an incomplete representation of survival patterns, particularly regarding short‐ and long‐term behavior in their review of the NEOCRTEC5010 statistics. In contrast, RMST provided an inclusive summary of the survival curve, incorporating all survival information up to the restricted time frame. In the present study, survival decreased sequentially in ypT0N0, ypT + N0, ypT0N+, and ypT + N+ patients. This classification system would provide clinicians with a highly discriminating and efficient method to assess and predict prognosis after NCRT.

We focused on the ypT0N+ patients. This specific subpopulation occupied a low prevalence of approximately 10% [22]. Until now, limited studies have reported the survival data for these patients and were controversial. Chao YK et al. [11] demonstrated that patients with ypT0N+ esophageal cancer had poorer survival rates than those with ypT0N0 (3‐year OS: 30.1% vs 55.9%, *p* < 0.001). Kim MP et al. [22] found that the survival rate of patients with ypT0N+ esophageal cancer was comparable to that of patients with pathologic partial response (pPR) and TNM stage II disease. However, Cho HJ et al. [12] showed no significant difference in OS and RFS between ypT0N+ and ypT0N0 patients. Additionally, the analysis of data from the NEOCRTEC5010 prospective clinical trial implies that persistent pathologic LN metastasis after NCRT is a critical factor in the poor prognosis of ESCC [23]. Overall, our findings were in agreement with the majority of studies that confirmed a significant decrease in both OS and RFS among patients with ypT0N+ tumors compared to those with ypT0N0 tumors. RFS did not differ statistically from that of the ypT + N+ group with an incomplete response.

Tumor recurrence and metastasis have a significant impact on the survival of patients [24]. Therefore, our study discussed the recurrence patterns of patients with different pathological regression types, with a special focus on the differences among ypT0N+ patients and other categories. Schroeder W et al. [25] compared the recurrence patterns between the ypT0N+ and ypT0N0 groups, and no significant difference was found. This is different from our findings, which showed that the LRR rate of the ypT0N+ group was significantly higher than that of the ypT0N0 group. Furthermore, the DM rate of the ypT0N+ group was similar to that of the ypT+N+ group and significantly higher than that of the ypN0 group. This may indicate that N+ patients have a considerably higher DM rate, irrespective of whether the primary lesions are in regression or not. Additionally, the OS of ypT0N+ patients did not exhibit statistical significance compared to that of T+N0 patients. However, we observed a distinction in the recurrence pattern, that ypT0N+ had a very similar LRR rate and a higher DM rate to that of ypT+N0. A study has shown that lymph node metastasis can promote distant metastasis. This is attributed to LN metastasis resisting T cell‐mediated cytotoxicity, inducing antigen‐specific regulatory T cells, and generating tumor‐specific immune tolerance, thereby facilitating distant tumor colonization [26]. Furthermore, some studies have suggested that tumor cells infiltrating the lymph node parenchyma can promote distant metastasis by invading the lymph node vasculature. Patients with positive lymph nodes on postoperative pathology may have circulating micrometastases, leading to a higher rate of distant metastasis [27, 28, 29]. A study [30] reported that metastatic lymph nodes have distinct biological characteristics compared to the primary lesion, suggesting that positivity in the primary lesion and lymph nodes may have different prognostic implications. Consistent with our findings, a Japanese study [31] analyzing recurrence patterns demonstrated that lymph node positivity is more strongly associated with distant metastasis compared to primary lesion positivity. In contrast, primary lesion positivity was significantly associated with local recurrence. These results suggest that patients with different pathological regression types may require different treatment options.

A recent real‐world study conducted at 33 centers worldwide investigated the potential benefits of adjuvant therapy for patients with esophageal cancer who underwent radical esophagectomy after neoadjuvant therapy and showed that patients with high nodal burden, ypT4, and positive margins may benefit the most [32]. Another study in the National Cancer Database indicated a similar result that patients with ypN+ disease who received adjuvant therapy had a 40% lower risk of death [33]. However, the initial review and meta‐analysis revealed no significant survival benefits in ypN+ patients, in contrast to a meaningful survival benefit in patients with mixed LN status. This may be explained by the fact that persistent LN‐positive disease does not respond well to any treatment regimen [34]. Rompen IF et al. [35] have shown that after neoadjuvant therapy, adjuvant therapy may have an age‐dependent benefit, with elderly patients being less likely to benefit from adjuvant treatment. The landmark Checkmate 577 study, in the field of immunotherapy for esophageal cancer, demonstrated a significant benefit of postoperative immune maintenance therapy in patients with NCRT and surgery for non‐pCR esophageal cancer. Nivolumab notably prolonged RFS and significantly reduced the incidence of distant metastases (6‐month DMFS: 78 vs. 71%, median DMFS: 28.3% vs. 17.6%) [36]. However, the efficacy of adjuvant therapy in patients with ypN+ remains uncertain, and further investigations are necessary to develop effective strategies to reduce the risk of DM in this patient population.

The current results showed that the majority of patients (93.9%) had recurrences within 3 years and 88.4% within 2 years. As a result, close monitoring of patients during the 2–3 year postoperative follow‐up may be critical. This finding is also supported by previous studies [37, 38]. Regional lymph nodes (38.9%) and lungs (25.0%) were the most frequent sites of recurrence among the patients. Consequently, chest CT should be used as a monitoring modality. We also explored the risk factors for recurrence and found that ypN+, ypT3‐4, and anastomotic fistula were independent risk factors for recurrence. This explains the significant difference in recurrence rates in patients between different types of pathological regression of the primary tumor and lymph nodes. Kofoed SC et al. [39] indicate that anastomotic fistula heightens the risk of recurrence, particularly distant recurrence, for reasons requiring further investigation. The incidence of anastomotic fistulas should be minimized. The radiation dose is also a risk factor for survival. Previous studies have shown that OS significantly worsens when the radiation dose is less than 40 Gy [40, 41]. Currently, multiple guidelines recommend a neoadjuvant radiation dose of greater than 40 Gy.

To our knowledge, this study presents the largest data on recurrence patterns in ESCC patients with pathology type ypT0N+ after neoadjuvant therapy and esophagectomy. However, this study has several limitations. First, the retrospective nature of the study may have significantly influenced the results due to selection bias. Second, patients from the three institutions were not treated simultaneously, potentially introducing additional biases. Additionally, discrepancies in the baseline characteristics among different groups, including tumor location and neoadjuvant chemotherapy, may have an impact on the analysis outcomes. Nonetheless, fewer than 10% (8.1%) of patients did not receive neoadjuvant chemotherapy. These factors were incorporated into our multivariable analysis and did not demonstrate any association with survival or recurrence.

## 5 Conclusion

Significant survival differences existed among patients with various types of pathological regression of the primary tumor and lymph nodes who received NCRT or NRT and surgery for ESCC. The ypT0N+ group had a poorer survival rate and higher DM rate than the ypT0N0 group. For these lymph node‐positive patients, adjuvant chemotherapy does not appear to improve prognosis.

## Author Contributions

**Fangdong Zhao:** visualization (equal), writing – original draft (equal). **Qifeng Wang:** data curation (equal). **Weiming Han:** data curation (equal). **Wubulaishan Maitudi:** formal analysis (equal). **Tian Zhang:** conceptualization (equal). **Xi Chen:** methodology (equal). **Jie Dong:** formal analysis (equal). **Fuliang Cao:** investigation (equal), resources (equal). **Lei Gong:** resources (equal), supervision (equal). **Xiaobin Shang:** data curation (equal). **Hongjing Jiang:** resources (equal), supervision (equal). **Wencheng Zhang:** methodology (equal), project administration (equal), resources (equal). **Qingsong Pang:** project administration (equal), resources (equal). **Ping Wang:** project administration (equal), resources (equal). **Zefen Xiao:** data curation (equal). **Peng Tang:** resources (equal), supervision (equal).

## Ethics Statement

The study was approved by the ethics committee of Tianjin Medical University Cancer Institute and Hospital (bc2023183). Since it is a retrospective study that presents no risks to the participants' health or economic well‐being, the ethics committee of Tianjin Medical University Cancer Institute and Hospital granted an exemption from obtaining informed consent. All the methods performed in this study were in accordance with the guidelines stated in the “Ethics Approval and Consent to Participate” statement provided above.

## Consent

All authors approve the manuscript for publication.

## Conflicts of Interest

The authors declare no conflicts of interest.

## Supporting information


**Figure S1.** The relationship between preoperative biopsy pathology and postoperative pathology.

**Figure S2.** Recurrence time of ypN0 and ypN+ patients.

**Figure S3.** Overall survival (A) and recurrence‐free survival (B) of esophageal squamous cell carcinoma patients with different pathologic regression types after 2012.

**Figure S4.** Restricted mean overall survival time (A) and restricted mean recurrence‐free survival time(B) of esophageal squamous cell carcinoma patients with different pathologic regression types after 2012. From left to right, the order is ypT0N0, ypT + N0, ypT0N+, ypT + N+.

**Figure S5.** Locoregional failure‐free survival (A) and distant metastasis‐free survival (B) of esophageal squamous cell carcinoma patients with different pathologic regression types after 2012.

**Table S1.** OS and RFS Rate of Patients with Different Pathologic Regression Types.

**Table S2.** Patient Characteristics after 2012.

**Table S3.** Comparison of Recurrence Patterns in Different Pathologic Regression Types after 2012.

**Table S4.** OS and RFS Rate of Patients With Different Pathologic Regression Types after 2012.

**Table S5.** Patient Characteristics after PSM.

## Data Availability

Data and materials related to this work are available upon request.
