# Supplementary figures and images for: Multi‐Institutional Analysis of Survival and Recurrence Patterns of Different Pathological Regression Types After Neoadjuvant Chemoradiotherapy or Radiotherapy for Esophageal Squamous Cell Carcinoma
Source: Cancer Med. 2025 Feb 13;14(4):e70676. doi: 10.1002/cam4.70676 (PMC11822455; doi:10.1002/cam4.70676)

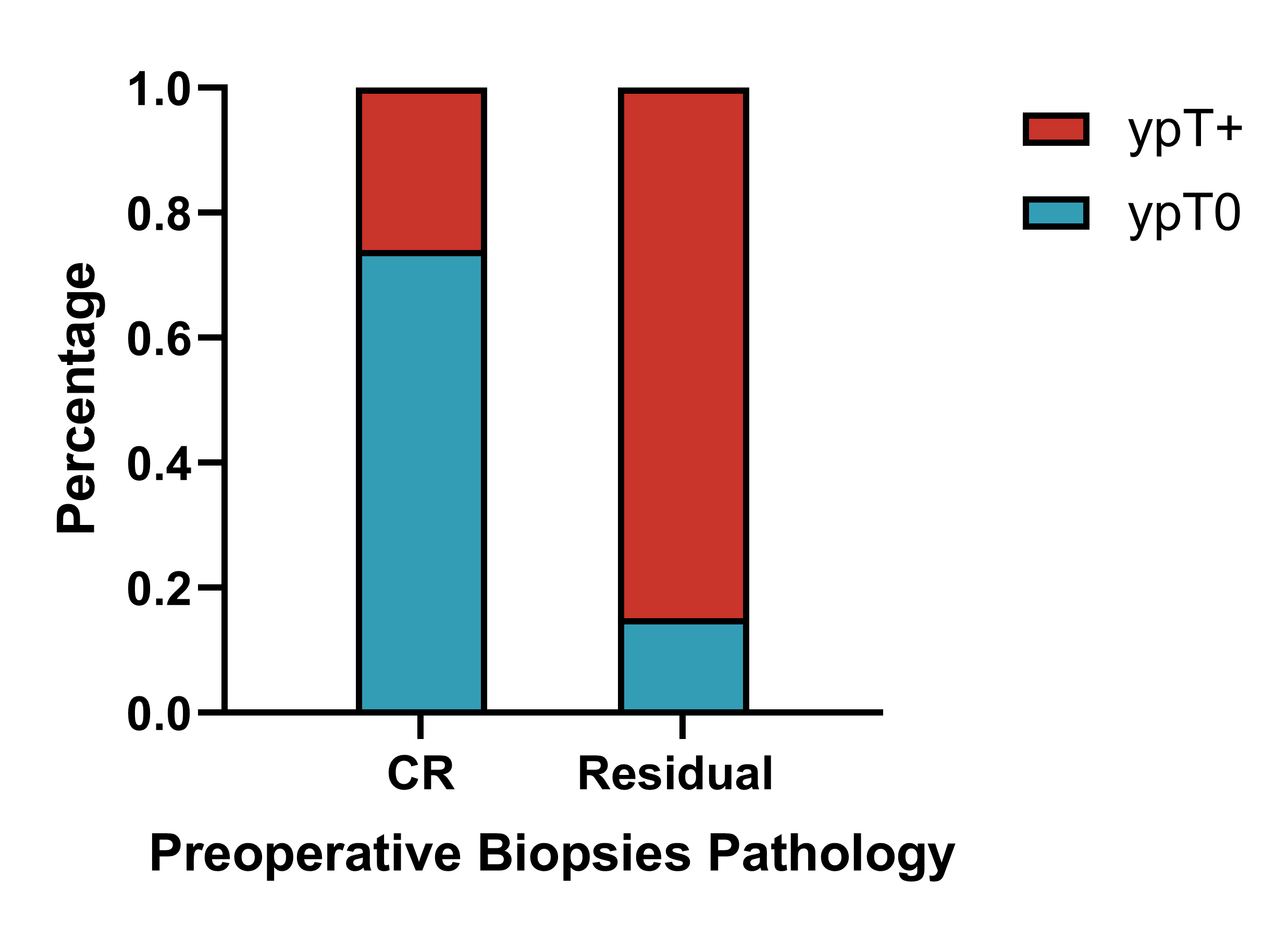

Supplement: Supplementary file 1 — Figure S1. The relationship between preoperative biopsy pathology and postoperative pathology. [file CAM4-14-e70676-s006.tif]

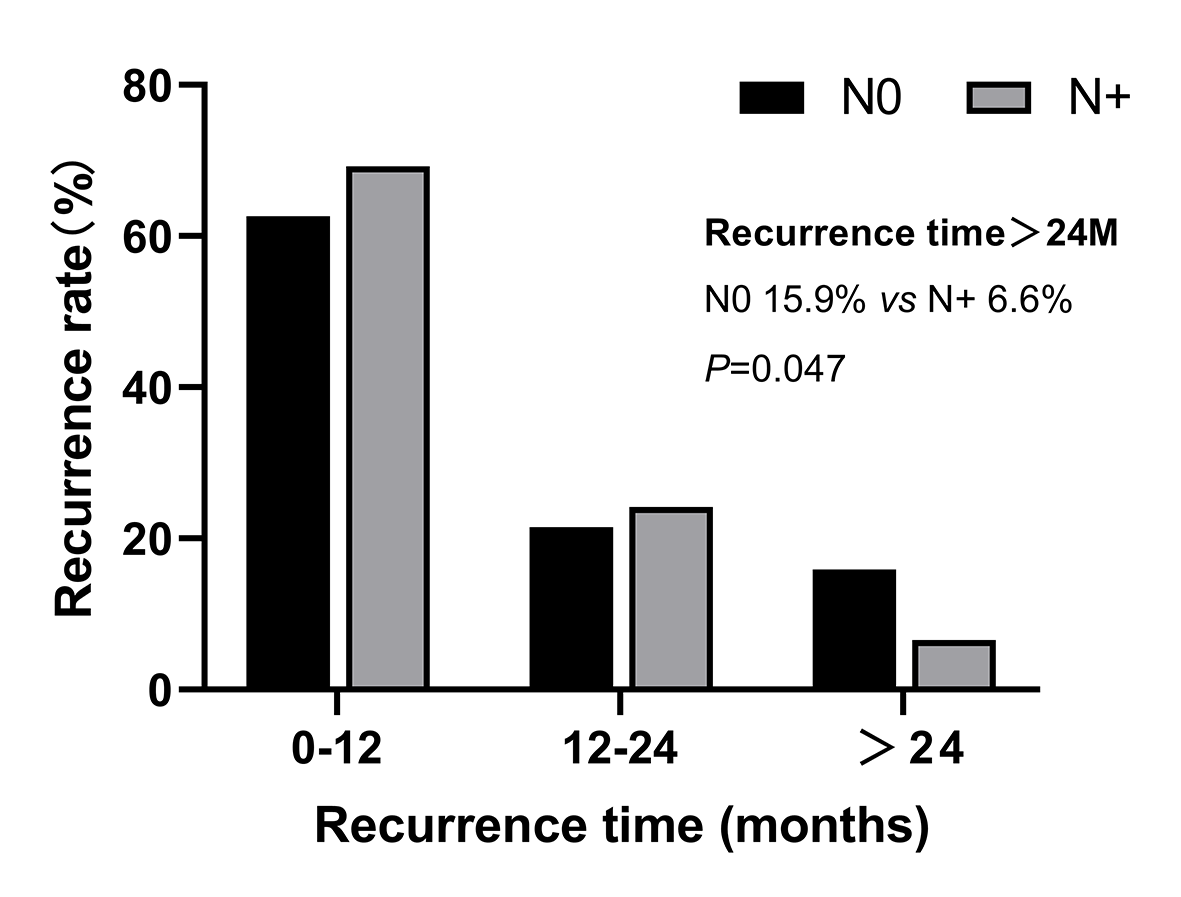

Supplement: Supplementary file 2 — Figure S2. Recurrence time of ypN0 and ypN+ patients. [file CAM4-14-e70676-s010.tif]

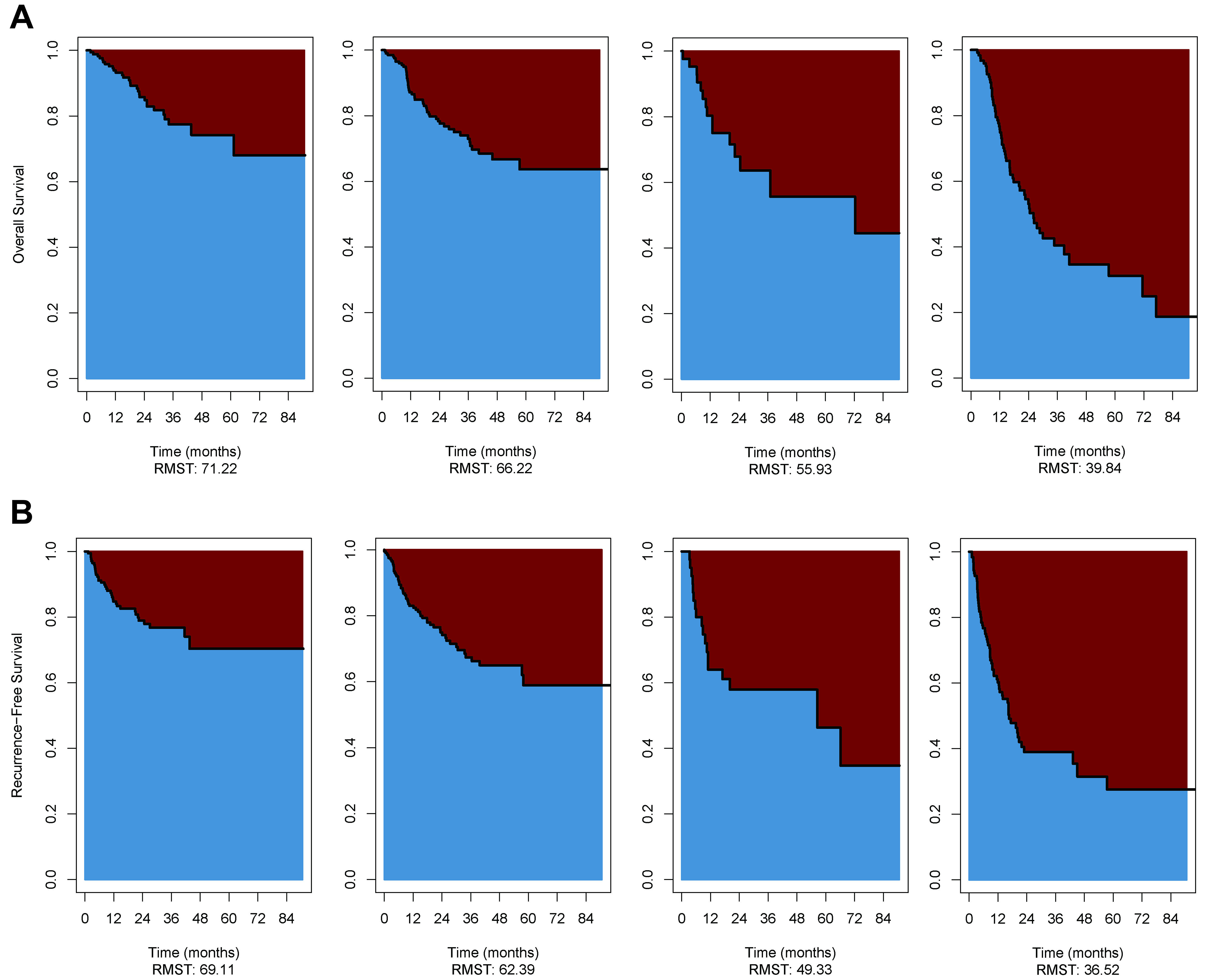

Supplement: Supplementary file 4 — Figure S4. Restricted mean overall survival time (A) and restricted mean recurrence‐free survival time(B) of esophageal squamous cell carcinoma patients with different pathologic regression types after 2012. From left to right, the order is ypT0N0, ypT + N0, ypT0N+, ypT + N+. [file CAM4-14-e70676-s009.tif]
