# Supplementary material for: Multi‐Institutional Analysis of Survival and Recurrence Patterns of Different Pathological Regression Types After Neoadjuvant Chemoradiotherapy or Radiotherapy for Esophageal Squamous Cell Carcinoma
Source: Cancer Med. 2025 Feb 13;14(4):e70676. doi: 10.1002/cam4.70676 (PMC11822455; doi:10.1002/cam4.70676)
Supplement: Supplementary file 6 — Table S1. OS and RFS Rate of Patients with Different Pathologic Regression Types. [file CAM4-14-e70676-s007.docx]

Supplemental Table 1. OS and RFS Rate of Patients with Different Pathologic Regression Types

|  | 1-Year OS (95% CI) | 3-Year OS (95% CI) | 5-Year OS (95% CI) |
| --- | --- | --- | --- |
| ypT0N0 | 93.1(89.3-96.9) | 75.7(68.3-83.9) | 73.1(64.5-82.7) |
| ypT+N0 | 85.0(80.5-89.9) | 68.4(61.8-75.7) | 61.4(53.5-70.4) |
| ypT0N+ | 81.7 (71.0-94.0) | 63.7 (49.7-81.6) | 57.3 (41.5-79.2) |
| ypT+N+ | 76.8 (69.7-84.6) | 41.8 (32.7-53.5) | 29.8 (20.2-44.0) |
|  | 1-Year RFS (95% CI) | 3-Year RFS (95% CI) | 5-Year RFS (95% CI) |
| ypT0N0 | 85.0 (79.8-90.5) | 76.0 (69.3-83.3) | 68.8 (59.4-79.7) |
| ypT+N0 | 81.0 (76.0-86.5) | 66.3 (59.5-73.8) | 60.1 (51.7-69.8) |
| ypT0N+ | 64.1 (51.1-80.5) | 55.8 (42.4-73.6) | 46.5 (29.6-73.1) |
| ypT+N+ | 61.5 (53.4-70.7) | 39.0 (30.4-50.1) | 29.5 (19.6-44.2) |

Data are presented as %.

OS, overall survival; RFS, recurrence-free survival; CI, confidence interval.
