# Supplementary material for: Multi‐Institutional Analysis of Survival and Recurrence Patterns of Different Pathological Regression Types After Neoadjuvant Chemoradiotherapy or Radiotherapy for Esophageal Squamous Cell Carcinoma
Source: Cancer Med. 2025 Feb 13;14(4):e70676. doi: 10.1002/cam4.70676 (PMC11822455; doi:10.1002/cam4.70676)
Supplement: Supplementary file 7 — Table S2. Patient Characteristics after 2012. [file CAM4-14-e70676-s004.docx]

Supplemental Table 2. Patient Characteristics after 2012

| Characteristics | ypT0N0  (n=169) % | ypT+N0  (n=201) % | ypT0N+  (n=42) % | ypT+N+  (n=122) % | P-Value |
| --- | --- | --- | --- | --- | --- |
| Age  ＜60  ≥60 | 74（43.7）  95（56.3） | 91（45.3）  110（54.7） | 23（54.8）  19（45.2） | 67（54.9）  55（45.1） | 0.179 |
| Sex  Male  Female | 141（83.5）  28（16.5） | 175（87.1）  26（12.9） | 36（85.7）  6（14.3） | 113（92.6）  9（7.4） | 0.145 |
| Tumor location  Upper  Middle  lower | 28（16.5）  82（48.6）  59（34.9） | 33（16.4）  80（39.8）  88（43.8） | 7（16.7）  11（26.2）  24（57.1） | 10（8.2）  55（45.1）  57（46.7） | 0.030 |
| Clinical T stage  T1-2  T3  T4 | 10（5.9）  119（70.5）  40（23.6） | 12（6.0）  124（61.7）  65（32.3） | 2（4.8）  29（69.0）  11（26.2） | 7（5.8）  77（63.1）  38（31.1） | 0.665 |
| Clinical N stage  N0  N1 | 13（7.7）  156（92.3） | 16（8.0）  185（92.0） | 2（4.8）  40（95.2） | 7（5.7）  115（94.3） | 0.600 |
| Clinical TNM stage  IIA-IIB  III  IVA-IVB | 16（9.4）  149（88.2）  4（2.4） | 19（9.6）  181（90.0）  1（0.4） | 4（9.5）  38（90.5）  0（0） | 11（9.0）  109（89.4）  2（1.6） | 0.857 |
| Radiation dose  ≤40Gy  ＜40Gy | 121（71.6）  48（28.4） | 155（77.1）  46（22.9） | 36（85.7）  6（4.3） | 100（82.0）  22（18.0） | 0.097 |
| Chemotherapy  Yes  No | 163（96.4）  6（3.6） | 190（94.5）  11（5.5） | 42（100.0）  0（0.0） | 112（91.8）  10（8.2） | 0.146 |
| Number of LN examined  ＜10  ≥10 | 15（8.9）  154（91.1） | 26（12.9）  175（87.1） | 1（2.4）  41（97.6） | 10（8.2）  112（91.8） | 0.156 |

LN, lymph nodes.
