# Supplementary material for: Multi‐Institutional Analysis of Survival and Recurrence Patterns of Different Pathological Regression Types After Neoadjuvant Chemoradiotherapy or Radiotherapy for Esophageal Squamous Cell Carcinoma
Source: Cancer Med. 2025 Feb 13;14(4):e70676. doi: 10.1002/cam4.70676 (PMC11822455; doi:10.1002/cam4.70676)
Supplement: Supplementary file 8 — Table S3. Comparison of Recurrence Patterns in Different Pathologic Regression Types after 2012. [file CAM4-14-e70676-s002.docx]

Supplemental Table 3. Comparison of Recurrence Patterns in Different Pathologic Regression Types after 2012

|  | Locoregional Recurrence | |  | Distant Metastasis | |
| --- | --- | --- | --- | --- | --- |
|  | **HR（95%CI）** | **P-Value** |  | **HR（95%CI）** | **P-Value** |
| T0N0 vs T0N+ | 2.587（0.867-7.722） | 0.088 |  | 2.081（1.019-4.253） | 0.044 |
| T0N+ vs T+N+ | 2.406（0.933-6.205） | 0.069 |  | 1.002（0.496-2.022） | 0.997 |
| T+N0 vs T0N+ | 0.828（0.323-2.121） | 0.694 |  | 2.475（1.212-5.057） | 0.013 |
| T0N0 vs T+N0 | 3.126（1.495-6.533） | 0.002 |  | 0.841（0.477-1.481） | 0.549 |

HR, hazard ratio; CI, confidence interval.
