# Supplementary material for: Multi‐Institutional Analysis of Survival and Recurrence Patterns of Different Pathological Regression Types After Neoadjuvant Chemoradiotherapy or Radiotherapy for Esophageal Squamous Cell Carcinoma
Source: Cancer Med. 2025 Feb 13;14(4):e70676. doi: 10.1002/cam4.70676 (PMC11822455; doi:10.1002/cam4.70676)
Supplement: Supplementary file 9 — Table S4. OS and RFS Rate of Patients With Different Pathologic Regression Types after 2012. [file CAM4-14-e70676-s008.docx]

Supplemental Table 4. OS and RFS Rate of Patients With Different Pathologic Regression Types after 2012

|  | 1-Year OS (95% CI) | 3-Year OS (95% CI) | 5-Year OS (95% CI) |
| --- | --- | --- | --- |
| ypT0N0 | 93.9(90.3-97.6) | 77.4(69.8-85.9) | 74.2(65.0-84.8) |
| ypT+N0 | 87.1(82.5-92.0) | 73.0(66.2-80.4) | 63.7(54.5-74.4) |
| ypT0N+ | 80.3 (69.0-93.6) | 63.6 (49.0-82.5) | 55.6 (38.4-80.5) |
| ypT+N+ | 75.8 (68.4-84.1) | 40.4 (30.9-52.9) | 31.1 (20.8-46.6) |
|  | 1-Year RFS (95% CI) | 3-Year RFS (95% CI) | 5-Year RFS (95% CI) |
| ypT0N0 | 84.7 (79.4-90.4) | 76.8 (70.0-84.2) | 70.3 (60.3-82.1) |
| ypT+N0 | 83.0 (77.9-88.5) | 67.4 (60.1-75.5) | 58.9 (40.9-70.8) |
| ypT0N+ | 64.0 (50.5-81.1) | 57.9 (44.0-76.2) | 46.3 (27.6-77.7) |
| ypT+N+ | 60.2 (51.8-69.9) | 38.9 (30.0-50.6) | 27.5 (17.1-44.3) |

Data are presented as %.

OS, overall survival; RFS, recurrence-free survival; CI, confidence interval.
