# Supplementary material for: Multi‐Institutional Analysis of Survival and Recurrence Patterns of Different Pathological Regression Types After Neoadjuvant Chemoradiotherapy or Radiotherapy for Esophageal Squamous Cell Carcinoma
Source: Cancer Med. 2025 Feb 13;14(4):e70676. doi: 10.1002/cam4.70676 (PMC11822455; doi:10.1002/cam4.70676)
Supplement: Supplementary file 10 — Table S5. Patient Characteristics after PSM. [file CAM4-14-e70676-s003.docx]

Supplemental Table 5. Patient Characteristics after PSM

| **Characteristics** | **No adjuvant treatment** | **Adjuvant treatment** | **P-value** |
| --- | --- | --- | --- |
|  | **(N=70)** | **(N=71)** |  |
| Age |  |  | 0.804 |
| ＜60 | 36 (51.4%) | 39 (54.9%) |  |
| ≥60 | 34 (48.6%) | 32 (45.1%) |  |
| Sex |  |  | >0.999 |
| Male | 64 (91.4%) | 64 (90.1%) |  |
| Female | 6 (8.6%) | 7 (9.9%) |  |
| Clinical T stage | |  | >0.999 |
| T1-2 | 2 (2.9%) | 3 (4.2%) |  |
| T3-4 | 68 (97.1%) | 68 (95.8%) |  |
| Clinical N stage | |  | >0.999 |
| N0 | 9 (12.9%) | 9 (12.7%) |  |
| N1 | 61 (87.1%) | 62 (87.3%) |  |
| Tumor location | |  | 0.943 |
| Upper/Middle | 30 (42.9%) | 29 (40.8%) |  |
| lower | 40 (57.1%) | 42 (59.2%) |  |
| Radiation dose | |  | >0.999 |
| ≤40Gy | 65 (92.9%) | 66 (93.0%) |  |
| ＞40Gy | 5 (7.1%) | 5 (7.0%) |  |
| Chemotherapy | |  | >0.999 |
| Yes | 1 (1.4%) | 1 (1.4%) |  |
| No | 69 (98.6%) | 70 (98.6%) |  |
| Number of LN examined | | | 0.579 |
| ＜10 | 5 (7.1%) | 8 (11.3%) |  |
| ≥10 | 65 (92.9%) | 63 (88.7%) |  |
| yp T stage |  |  | 0.941 |
| T0-2 | 38 (54.3%) | 40 (56.3%) |  |
| T3 | 25 (35.7%) | 25 (35.2%) |  |
| T4 | 7 (10.0%) | 6 (8.5%) |  |
| Clinical N stage | |  | 0.601 |
| N0 | 42 (60.0%) | 38 (53.5%) |  |
| N1 | 20 (28.6%) | 21 (29.6%) |  |
| N2-3 | 8 (11.4%) | 12 (16.9%) |  |
| Vessel carcinoma embolus | | | 0.725 |
| No | 64 (91.4%) | 67 (94.4%) |  |
| Yes | 6 (8.6%) | 4 (5.6%) |  |
